# Diffusion and perfusion MRI of normal, preeclamptic and growth-restricted mice models reveal clear fetoplacental differences

**DOI:** 10.1038/s41598-020-72885-9

**Published:** 2020-10-02

**Authors:** Qingjia Bao, Ron Hadas, Stefan Markovic, Michal Neeman, Lucio Frydman

**Affiliations:** 1 Department of Chemical and Biological Physics, Weizmann Institute, 7610001 Rehovot, Israel; 2 Department of Biological Regulation, Weizmann Institute, 7610001 Rehovot, Israel

**Keywords:** Developmental biology, Intrauterine growth, Animal disease models, Development of the nervous system, Preclinical research

## Abstract

Diffusion-weighted MRI on rodents could be valuable to evaluate pregnancy-related dysfunctions, particularly in knockout models whose biological nature is well understood. Echo Planar Imaging’s sensitivity to motions and to air/water/fat heterogeneities, complicates these studies in the challenging environs of mice abdomens. Recently developed MRI methodologies based on SPatiotemporal ENcoding (SPEN) can overcome these obstacles, and deliver diffusivity maps at ≈150 µm in-plane resolutions. The present study exploits these capabilities to compare the development in wildtype vs vascularly-altered mice. Attention focused on the various placental layers—deciduae, labyrinth, trophoblast, fetal vessels—that the diffusivity maps could resolve. Notable differences were then observed between the placental developments of wildtype vs diseased mice; these differences remained throughout the pregnancies, and were echoed by perfusion studies relying on gadolinium-based dynamic contrast-enhanced MRI. Longitudinal monitoring of diffusivity in the animals throughout the pregnancies also showed differences between the development of the fetal brains in the wildtype and vascularly-altered mice, even if these disparities became progressively smaller as the pregnancies progressed. These results are analyzed on the basis of the known physiology of normal and preeclamptic pregnancies, as well as in terms of the potential that they might open for the early detection of disorders in human pregnancies.

## Introduction

Understanding the fetoplacental unit, its functional dynamics and development, requires characterizing the transport of fluids within and between maternal, placental and fetal compartments^1,2^. Improvements in this understanding rest heavily on animal model research, which can be examined with a flexibility unavailable in human investigations^3^. Ex vivo anatomical investigations of rodents have proven valuable for unraveling essential structural details about the confines of these dynamic processes^4–6^, yet in vivo analyses are no less fundamental for connecting such morphologies with metabolic and transport phenomena, and their influence on health and disease^4,7^. Nuclear magnetic resonance imaging (NMR,MRI) offers a natural approach for achieving this, as it provides the means to measuring diffusive^8,9^ and perfusive^10,11^ transport of fluids and nutrients, and for spatially localizing these dynamics throughout the developmental process. Perfusion is usually associated with the active flow of biological fluids, and can be measured by the addition of an exogenous agent changing certain aspects of the NMR/MRI signature—for instance, the water’s T_1_ relaxation time. As this contrast agent is carried by the circulatory system and its influence is mapped, a 3D description reflecting the hemodynamics of a tissue will arise. Apparent diffusion coefficient (ADC) measurements complement this information, with a report on water’s capacity to spontaneously translate within an organ. These pseudo-random molecular motions, often influenced by unresolved intravoxel circulation, will in turn be dependent on parameters such as cellularity and microvascularity; NMR allows their non-invasive mapping throughout a tissue with the aid of pulsed-gradient spin-echo sequences^8–13^. The diffusion-weighted imaging (DWI) data leading to the ensuing ADC maps typically have to rely on single-shot echo-planar-imaging (EPI) sequences—more resilient than normal multi-shot MRI schemes to motion-derived artifacts^14,15^– as part of the measurement process. EPI, however, is sensitivite to field inhomogeneities, to water/fat interferences and to other non-idealities, that often prevents it from delivering information in the challenging conditions placed by the abdomens of pregnant, living rodents. This has led to a number of alternative proposals that rely on multiple pulses to overcome these distortions^4,16,17^; we have shown that spatiotemporal encoding (SPEN) techniques^18–20^ have the potential to deal with these limitations. Further, although initial single-shot SPEN studies suffered from a limited sensitivity that constrained its DWI usage to the study of pregnant rats^19^, we have recently developed optimized interleaved approaches that can deliver diffusivity maps with ~ 100 µm in-plane resolutions^20,21,22^—sufficient to provide detailed ADC images of living, pregnant mice.

The aim of this study was to explore the insight provided by these new methods when used to monitor the progress of fetoplacental units from day E14.5 onwards, in studies comparing the development of wildtype and of vascularly-altered mice. The latter were assessed for two kinds of knock-out mice models: eNOS (endothelial nitric oxide synthase) deficient (−/−) animals, which are associated with intrauterine growth-restriction (IUGR) symptoms^23,24^, and IL10 knockout mice, exhibiting hypertension and proteinuria during pregnancy and serving as models for preeclampsia (PE,^25,26^). These knockout mice measurements were complemented by studying a third group of mice treated with Nω-Nitro-l-arginine methyl ester hydrochloride (l-NAME), a chemical known to induce hypertension, proteinuria and decreased fetal / litter size—all these symptoms often observed in PE pregnancies as well^27^. The ADC maps of several fetoplacental compartments could be clearly discerned by SPEN DWI; these measurements were used to longitudinal follow the development of placentas and fetal brains throughout the various models. The average ADC distributions adopted throughout the maternal and fetal layers of the placentas of the IUGR and PE models were substantially different to those of healthy animals—both in their average values and in their distributions. Related effects were also noted when performing measurements with gadolinium-based T1 contrast agents, which showed clear differences in perfusion between the placentas of these different animals, as well as between the behavior of certain fetal organs. The development and arguably maturation of fetal brains, at least as measured by morphology and by the changes in the average ADCs observed with age, were remarkably constant for all the mice models. However, the distributions of the fetal brain ADCs in the MRI maps of the wildtype animals, were different from those in the diseased models. Potential rationalization of these observations on the bases of known physiological features associated to IUGR and PE, as well as their implications to human pregnancies, are briefly discussed.

## Materials and methods

All methods in this study were carried out in accordance with relevant institutional guidelines and regulations.

### Animals

Animal experiments were preapproved by the Weizmann Institute IACUC system, which is fully accredited by the AAALAC, the US NIH Office of Laboratory Animal Welfare, and the Israel Ministry of Health. For the wildtype animal ADC mapping experiments, 5 female mice (C57BL/6 J, aged 8–10 weeks) were mated, and the detection of a vaginal plug the following day at noon was considered to be gestation day E0.5. These animals were scanned from days E10.5 to delivery; the bulk of this study focuses on ages E14.5–E19.5. For the eNOS^−/−^ and IL10^−/−^ knockout experiments n = 5 and n = 4 homozygous mice were mated, respectively; as for the wildtype conterparts, the presence of a copulation plug was denoted as day E0.5 of pregnancy, and scans on them were performed from E14.5 to E19.5. The mean weight of eNOS^−/−^ neonates after birth was ~ 10% lower than that of wild type animals ($1.17\pm 0.06$ vs. $1.32\pm 0.05$ g respectively), while the mean weight of the IL10^−/−^ pups was ~ 14% lower (1.12 g$\pm 0.04$). ADC mapping experiments were also carried out on l-NAME-treated pregnant mice (mean neonatal offspring weight 1.09 ± 0.1 g); to generate these, a daily dose of 50 mg/kg body weight of l-NAME in PBS (Sigma-Aldrich) was administered to four wildtype mice either intravenously or subcutaneously on days E14.5 and E15.5; these animals were scanned on day E14.5 before the first injection, and on day E16.5 after being subject to the two injections. In this l-NAME-injected group one mouse was excluded due to premature delivery (final n = 3).

A separate set of animals was subjected to dynamic contrast-enhanced (DCE) MRI involving the injection of Gadolinium-DTPA, a usual MRI contrast agents. DCE MRI was also assessed for Gd-DTPA linked to BSA, a high molecular weight protein that prevents extravasation of the contrast from the maternal to the fetal compartments^28^. In these experiments a catheter was placed in the animal’s tail vein, and pre-tested for intravascular perfusion using a 100 μl saline bolus (syringe size: 1 mL; catheter length: 70 cm; catheter diameter: 0.28 mm; dead volume from needle to tip: 43 μl; needle size: 30G). Doses of approximately 0.16 mmol/kg were used for the ≈30 g pregnant mice; this is a relatively low dose, but it was sufficient for imaging fetoplacental perfusion thanks to these organs’ high vascularization (higher dosages caused adverse effects, including signal cancellations due to high contrast agent concentration). These experiments were performed on pregnant mice at days E14.5 (for the wildtype and knockouts) or E16.5 (for the l-NAME-injected mice), using n = 3 wildtype mice for control (C57BL/6 J), n = 3 eNOS^−/−^ mice , n = 3 IL10^−/−^ mice, and n = 2 l-NAME-injected mice. All the wildtype mice supported well the injection of Gd-DTPA and gave birth normally; by contrast one eNOS^−/−^ , one IL10^−/−^ and two l-NAME-injected mice, had premature deliveries. Table 1 summarizes the mice/fetoplacental units analyzed in this study for the various experiments.

**Table 1:** Summary of animals and fetoplacental units analyzed by the experiments in this study.

Experiments and repetitions	Wildtype mice	eNOS^−/−^ mice	IL10^−/−^ mice	l-NAME treated mice
Number of animals—ADC analyses	5	5	5	3
Total fetoplacental units—ADC analyses^a^	22–35	26–38	17–29	11
Number of animals—Gd analyses	3	3	3	2
Total fetoplacental units—Gd analyses	12	15	13	7

^a^The number of fetoplacental units decreased as the pregnancy of a given animal advanced, as fewer units could be captured by the coil’s field-of-view; hence the ranges in this row reflect the larger number of fetuses captured at E14.5 over their E19.5 counterparts. ADC and DCE analyses for l-NAME-treated mice were only performed at one or two pregnancy days, and hence the fixed number of units.

### Magnetic resonance imaging methods

All data were collected on a DD2 7 T/110 mm horizontal magnet scanner (Agilent Technologies, Santa Clara, CA) using a Millipede quadrature probe. During the scans, the pregnant mice were anesthetized with isoflurane (1–2%) via a vaporizer, and the animal’s body temperature was maintained constant by using a water-based heating system. Respiration was monitored via a pressure sensor (SA-II, Stony Brook, NY) and maintained at 30–50 breaths per minute. Shimming was executed for each animal utilizing a PRESS-based sequence focusing on the abdomen; typical linewidths of ≈ 100 Hz were achieved prior to beginning the examinations. For recording the anatomical reference images scanner-provided fast spin-echo (RARE) sequences were used, with the following parameters: TR/TE = 2000/48 ms, 8 slices of 1 mm thickness, echo train length = 8, two averages, scan time around 90 s with the respiratory trigger.

**Figure 1:**
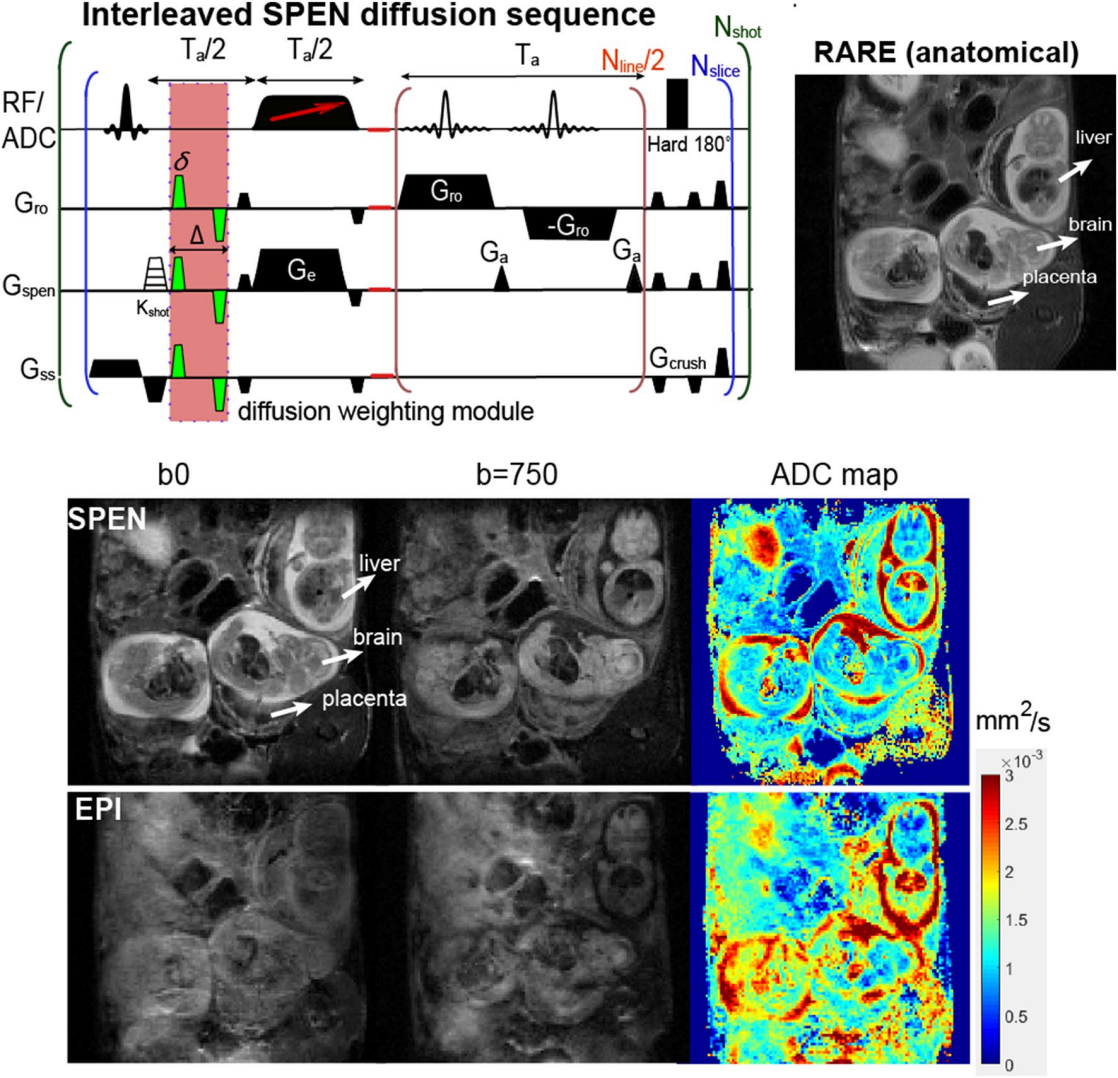
Multi-shot, fully-refocused, diffusion-weighted SPEN sequence (top left), and the comparison of high resolution b0 images, b-weighted images and ADC maps (bottom) obtained by SPEN and EPI for an eNOS^−/−^ mouse on day E14.5. Fetuses and their organs can be clearly visualized in the RARE image used as anatomical “gold standard” (top right), as well as in all the SPEN-derived data—but not so on the EPI b-weighted images. Acquisition parameters Multishot EPI: TR/TE = 2000/30 ms, FOV = 35 × 30 mm, data matrix = 128 × 128, 4 interleaves, and 4 averages. Multishot SPEN: TR/TE = 2000/37 ms, FOV = 30 × 30 mm, data matrix = 160 × 160, 5 interleaves, 4 averages. Diffusion parameters: δ = 3.2 ms, ∆ = 10 ms, diffusion gradient = 33G/cm (nominal b-value≈ 750 s/mm^2^), three orthogonal *G*_*d*_ orientations.

SPEN DWI sequences were ran within Agilent’s VNMRJ 3.2 software, using the multi-shot, multi-slice sequence shown in Fig. 1 (top left). The experiment’s principles are as described in Refs.^22,29^; they include a selective slice excitation, variable-orientation diffusion-weighting bipolar gradients placed within a full-refocusing period, an interleaved SPEN encoding module including an adiabatic 180° linearly swept pulse applied in the presence of an encoding gradient G_e_, an acquisition including an oscillating readout gradient scanning k_RO_ interspersed with blipped G_a_ gradients rasterizing the SPEN dimension, and a final hard 180° pulse to enable multi-slice acquisitions. Also included is a variable K_shot_ pulsed gradient along the SPEN direction, allowing for data interleaving and thereby for enhancing the spatial resolution. The sequence used—together with the necessary image processing and reconstruction scripts leading to the DWI data—are available at https://www.weizmann.ac.il/chemphys/Frydman_group/software for download. Typical abdominal SPEN scans involved the following parameters: TR/TE = 2000 ms/40 ms, Field-of-View (FOV) = 30 × 30 mm, in-plane nominal resolutions of 187 µm, four interleaved averages, 4–8 slices with 1 mm thickness, and respiration-triggered acquisitions. Higher resolution images (~ 140 µm nominal in-plane) were occasionally obtained by relying on SPEN’s “zooming” abilities and on reduced FOVs (23 × 23 mm). Full effective b-map calculations incorporating both these pulsed gradients as well as their cross-talk with the imaging gradients,^30^ were done to evaluate the results. Given SPEN’s reliance on relatively long (≈20 ms) encoding pulses applied in the present of an encoding gradient, minimal b-values in the present study were ca. 60 s/mm^2^, thereby preventing us from using its DWI for evaluating perfusive-like (IVIM)^13^ fluid motions.

DCE MRI^31^ data were collected using a gradient-echo (fast, low-angle shot, FLASH)^32^ sequence with the following parameters: TR/TE = 60/2.6 ms, 75° flip angle, matrix size = 128 $\times$ 128, 6 slices with resolution = 240 µm $\times$ 240 µm $\times$ 1 mm. The temporal resolution of these measurements was 7.7 s, and the data were acquired for 180 time points. Maps of the prefusivity rates were evaluated from these images using the steepest slope method^33,34^, an approach that quantifies perfusion based on the initial uptake phase of the contrast agent in the targeted organ. The steepest slope method affords an absolute quantification of a tissue’s perfusion in a computationally simple and numerically robust manner, but requires as previous definition an arterial input function; this was selected in every mouse based on the behavior observed on the well-perfused kidney hilus. Additional details regarding the scanning parameters are included in each figure and in its caption.

## Results

### ADC and perfusion measurements: general features

As mentioned, ADC maps can report on parameters such as cellularity and microvasculature. This in turn can help to better understand physiological parameters of relevance in order to evaluate, and eventually diagnose, pathological pregnancies: an increased cellularity for instance is characteristic of brain maturation^35^, whereas microvascularization is an indirect measure of a placenta’s ability to transport nutrients to and from the maternal–fetal interphase. When targeting structures like fetoplacental units in mice, however, this information is only meaningful if recorded at a suitably high quality and with sub-mm resolution. Figure 1 illustrates the advantages resulting upon deriving such high-quality, high-definition ADC maps using SPEN DWI, for an eNOS-defficient mouse in its E14.5 pregnancy day as case example. Presented in these panels are a multiscan RARE image serving as anatomical reference, as well as interleaved (multi-shot) SPEN and reversed-gradient EPI experiments collected on this animal in the absence (b0) and in presence of the bipolar diffusion-weighting module; also included are the ADC maps derived from these data. For the b0 images the quality of both SPEN and EPI data sets is comparable, yet upon activating the diffusion weighting only the former retain clear evidence of key fetal features including the heart, liver and brain. Also resolved in both the b0 and ADC maps are multiple placental layers, of the kind that have been recently observed in wildtype mice studies^22^. These layers involve a high-ADC region positioned further away from the fetus that is associated to the maternal decidua, a layer closer to the fetus that also has high ADC values which we associate to the placental labyrinth, and an intermediate pearled layer separating the two possessing the slowest ADC values, that we associate to the trophoblasts. Support for these compartments’ assignments can be gathered by T1-weighted experiments arising upon injecting BSA-GdDTPA into pregnant animals. BSA-GdDTPA is a high molecular weight contrast agent that shortens T1 thanks to the inclusion of gadolinium, but which is unable to cross the placental barrier due to the large size of the bovine serum albumin (BSA) protein that is associated to the metal. Supporting Figures S1a,b present such T1-weighted images collected before and after injection of BSA-GdDTPA on a dam at day E10.5; as can be appreciated, a placenta that is barely visible under such conditions before BSA-GdDTPA administration, becomes clearly visible thereafter. This is due to the high vascularity of this organ, which facilitates an efficient perfusion of the contrast agent. The placentas, however, are not uniformly highlighted by the Gd: the maternal labyrinth shows the highest contrast, partly highlighted is the pearled trophoblast cell structure, and revealing nearly no contrast are the fetal structures, which the protein-bound contrast agent cannot penetrate. Very similar placental layers are also highlighted by SPEN-derived ADC maps recorded on the same animal, as can be seen from Supporting Figures S1c,d.

**Figure 2:**
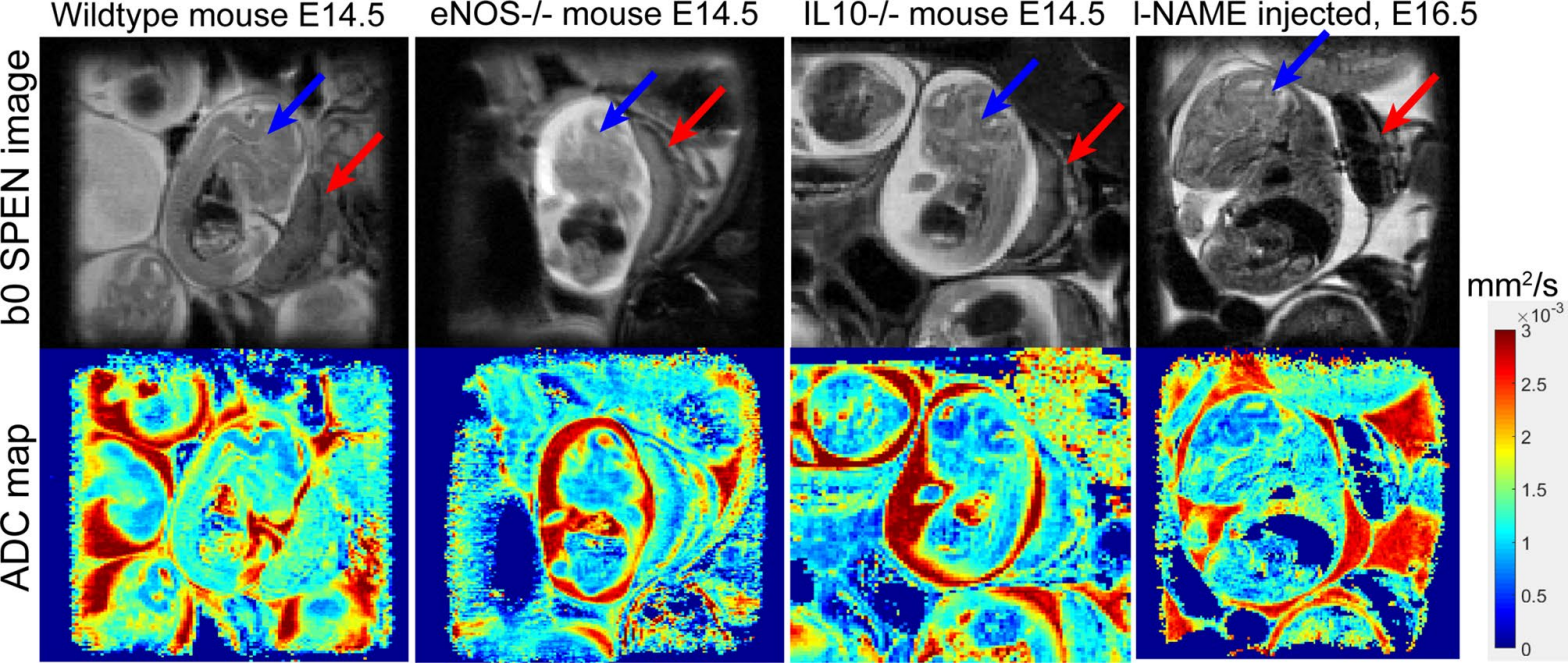
Coronal slices zooming into fetoplacental units, exemplifying the images and ADC maps derived from the multi-shot SPEN sequence in Fig. 1a, for different mice models. Data were collected on 1 mm slices with a 125 µm in-plane resolution using a 23 × 23 mm^2^ FOV. Additional parameters: TR/TE = 2000/32 ms, respiratory trigger, 5 interleaved shots, 8 averages per interleave, δ = 3.2 ms, ∆ = 10 ms, diffusion gradient = 33 G/cm (nominal b-value ≈ 750 s/mm^2^), three orthogonal directions, total scan time ~ 6 min. Red arrows highlight multi-layered placentas; blue arrows fetal brains; the rapidly-diffusing regions (intense red) in the ADC maps reflect the amniotic fluid. The l-NAME injected mouse was subject to two treatments prior to scanning on days E14.5 and E15.5, and hence E16.5 was the first day were differences versus wildtypes could be gathered.

With this as background, Fig. 2 summarizes the kind of diffusion-based measurements performed in this work: it shows b0 (anatomical) images of the animal models that were targeted, together with the corresponding SPEN-derived ADC maps. These images are zoom-ins into single fetoplacental regions of interest; many of which could be identified in each dam’s scan. These measurements were repeated longitudinally over the course of the pregnancy, in order to study how the diffusivity properties of the wildtype and diseased animal models varied over time. Although most of the images shown in this paper are illustrated with single-slice data, ADC fetoplacental maps were assessed in this study using multi-slice acquisitions—the need for multi-slicing arising from the demand to find the optimal positions that would allow us to target multiple fetoplacental organs for each dam throughout the analyses. Supporting Information Figures S2-S4 illustrate such representative volumetric sets collected on various animals.

### SPEN DWI—fetal brain development

**Figure 3:**
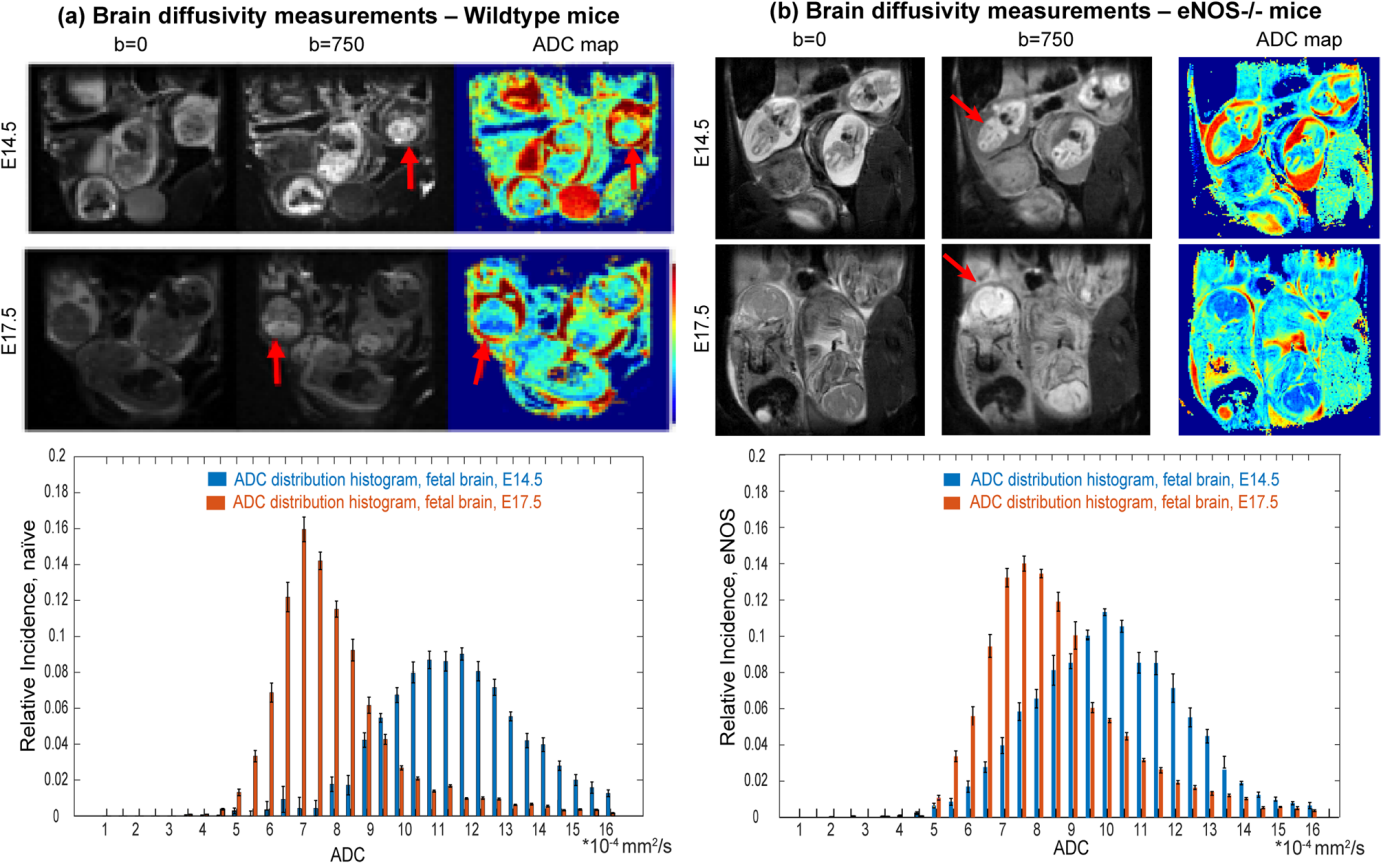
Top: In vivo coronal SPEN images focusing on the changes in ADCs experienced by fetal brains (exemplified by red arrows) in wildtype vs eNOS^−/−^ mice, for E14.5 and E17.5 days of pregnancy. Shown for each animal are b = 0 and 750 s/mm^2^ (nominal values) DWIs, as well as the derived isotropic ADC maps. FOVs and slice thicknesses for all the slices was 30 × 30 × 1 mm^3^; additional parameters: TR/TE 2000/42 ms, 5 interleaves, 4 averages. Bottom: ADC distributions that could be extracted from the images shown on top, for the different animals and ages. Error bars in the histograms (in black) reflect the distribution in the values as could be measured for the whole cadre of fetuses in all the animals.

**Figure 4:**
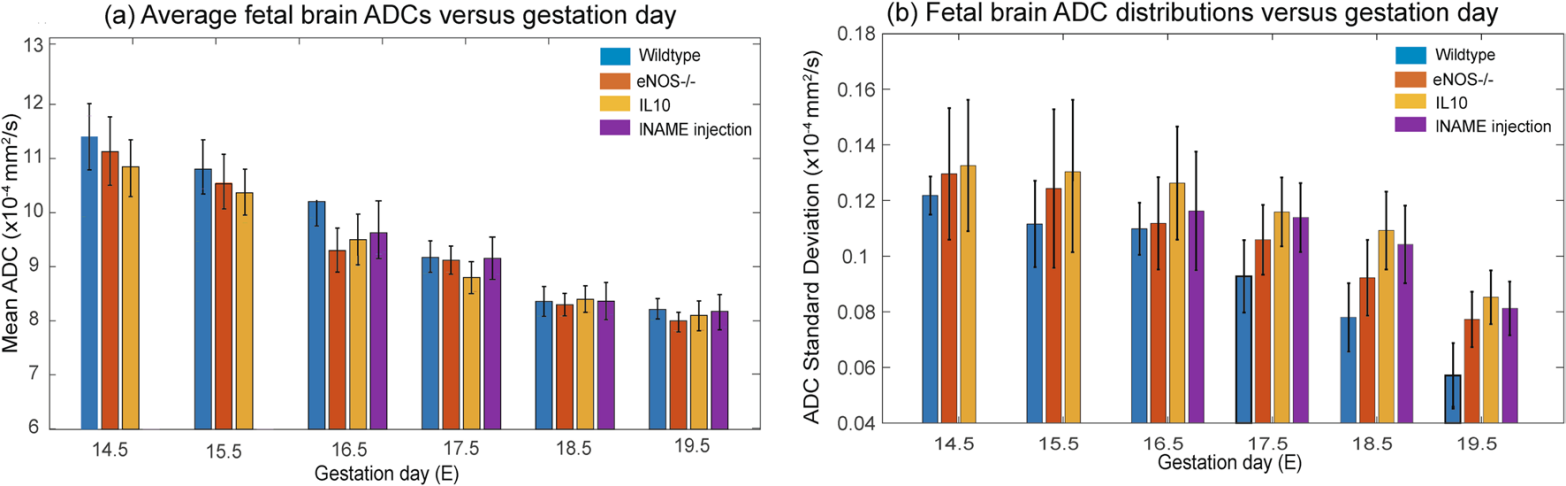
(**a**) Averages and (**b**) standard deviations of the ADC values measured in the fetal brains of the wildtype and knockout/l-NAME pregnant mice here studied. Results show average values arising from ~ 20 to 40 fetoplacental units (depending on the age and animal model) segmented as illustrated in this figure, and analyzed by Gaussian fittings. Error bars for each measurement represent the spread in average values detected for the mean and standard deviations of the ADCs over the full cohorts. All data were collected using the acquisition conditions described in Fig. 3.

As mentioned, water diffusivity can be used as aid for assessing brain maturation. SPEN-based DWI was thus used to explore whether differences in the apparent diffusivities emerged in the fetal brains, both among the various mice strains and as a function of their age. Figure 3 shows histograms of the ADC values measured for fetal brains in wildtype and eNOS^−/−^ animals at two different ages, together with representative MRI data sets of the kind that led to such results. A clear trend shown by these ADCs, as well as for IL10^−/−^ model (data not shown), is a progressive reduction in their average values with age. This behavior can be appreciated from the multi-day longitudinal progressions shown in Fig. 4a, which summarizes the average ADC values measured for the fetal brains in all of this study’s mice. This decrease in ADCs is as reported for healthy humans in general^36^. The histograms also evidence a certain bias of the overall ADCs in the wildtype mice towards higher brain diffusivities in early pregnancy stages, even if the average fetal brain ADCs in all animal classes end up reaching similar values by the pregnancy’s end. At this stage, however, significant differences could still be noticed in the distributions of ADCs within the fetal brains: this is conveyed in Fig. 4b, where histograms of the kind introduced in Fig. 3 for each age and fetoplacental unit in a given animal model, were analyzed under the assumption that they present a Gaussian distribution in their ADC values. The standard deviations of the resulting distributions narrow for all animals with age—but much more markedly for the wildtype mice than for the disease models.

### SPEN DWI—placental development

**Figure 5:**
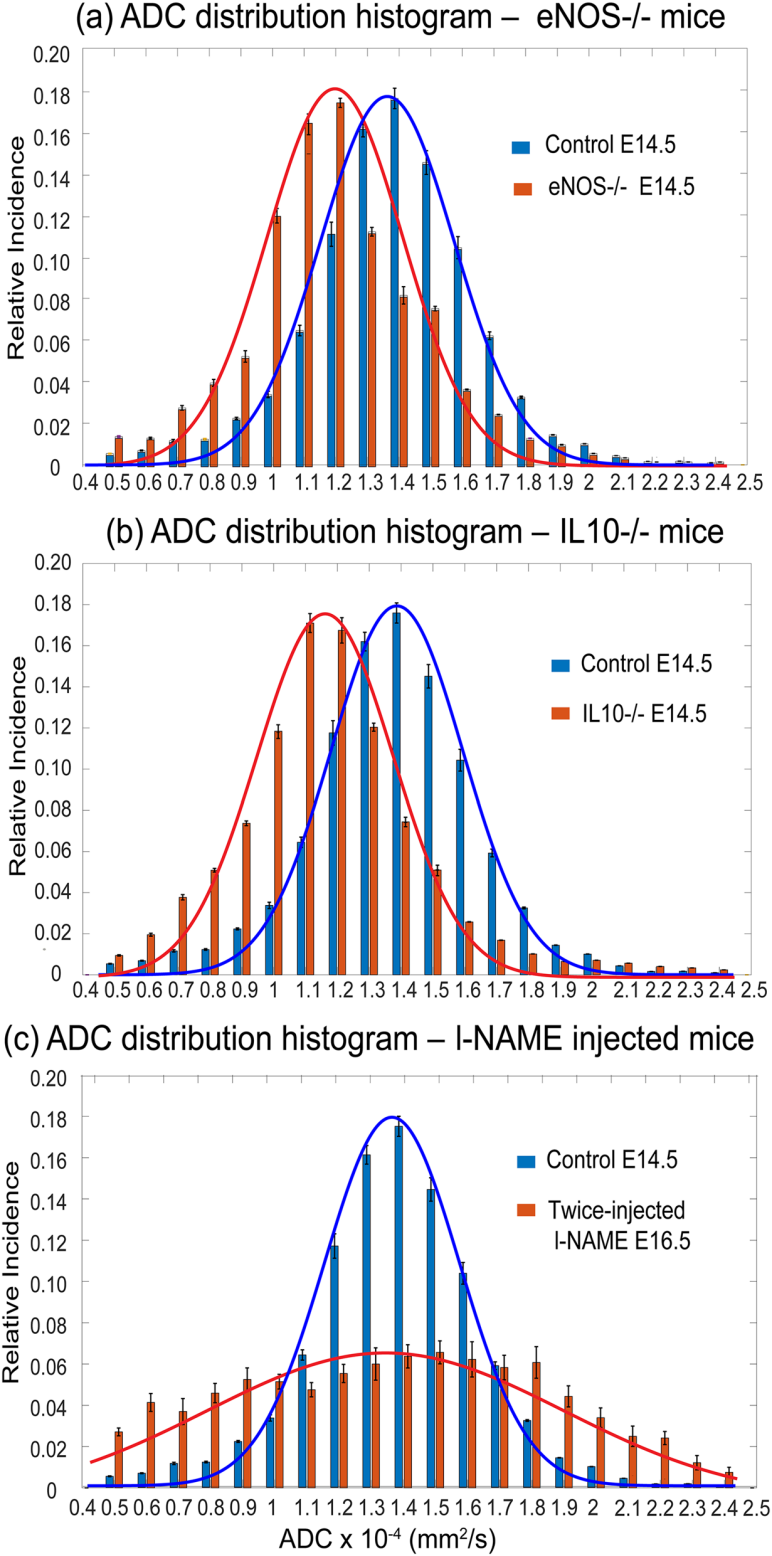
Representative histograms of ADC placental distributions for animals belonging to each of the various mice models hereby studied for day E14.5 (**a**,**b**); the data collected for the l-NAME-injected mice were collected on the same animal on E14.5, and two days after a double injection. The lines are continuous Gaussian fits to the histogram data, leading to average ADCs and their standard deviation distribution. Figure 6 summarizes this kind of analysis for all the animals and placentas analyzed in this work. All data arises from the acquisitions described in Fig. 3.

**Figure 6:**
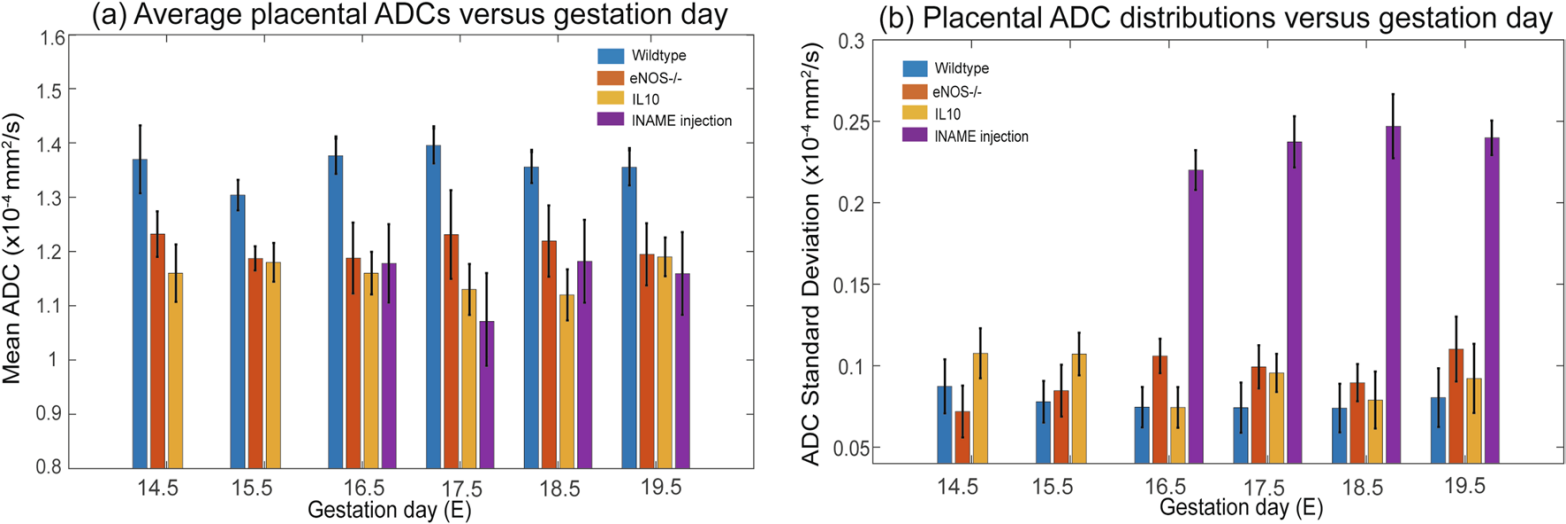
(**a**) Average placental ADC values for wildtype (control), eNOS^−/−^, IL10^−/−^ and l-NAME treated mice, plotted versus gestation day. (**b**) Distribution in the placental ADC values, as derived from histograms of the kind introduced in Fig. 5. The results presented arise from n = 5 wildtype mice (with a number of placentas 22–35 depending on the gestation day), n = 5 eNOS^−/−^ mice (number of placentas 26–38 depending on gestation day), n = 4 IL10^−/−^ mice (number of placentas is 17–29 depending on gestation day), and n = 3 l-NAME-treated mice (11 placentas). Error bars represent the spread in values observed for the mean and for the standard deviations of the reported ADCs over the full cohorts.

Figures 5 and 6 present a similar analysis as introduced above for brains, but focusing on how placental ADCs change in the wildtype, knockout, and chemically-induced vasocompromised models. By contrast to the brain-oriented studies, where diffusivity was associated with cellularity and maturation, the ADC values reflected by placentas is influenced by microvascularization—hence reporting on the organs’ transport capabilities. We have recently shown that placental ADCs remain relatively constant throughout the last week of naïve mice pregnancies^22^; average placental ADCs for the the knockout models also remain relatively constant yet at significantly lower average diffusivity values (Fig. 6a)—presumably reflecting the vascularization compromises in these models. These distinctions, however, are not evidenced on the knockout’s ADC distributions, which look relatively similar for these and for the wildtype mice. This is at variance with what is evidenced by the l-NAME-treated animals: although their average placental ADCs are not very different from their knockout counterparts, the l-NAME-treated ADC histogram distribution is considerably broader than all other counterparts (Fig. 6b). This could reflect an attempt of the physiology to overcome the onset of restriction diffusivities incurred on by the injection of the chemical, leading in turn to insufficient maternal/fetal transport of nutrients^27,37^, via the generation of faster-diffusing nutrition and irrigation paths.

### Perfusion MRI analyses

**Figure 7:**
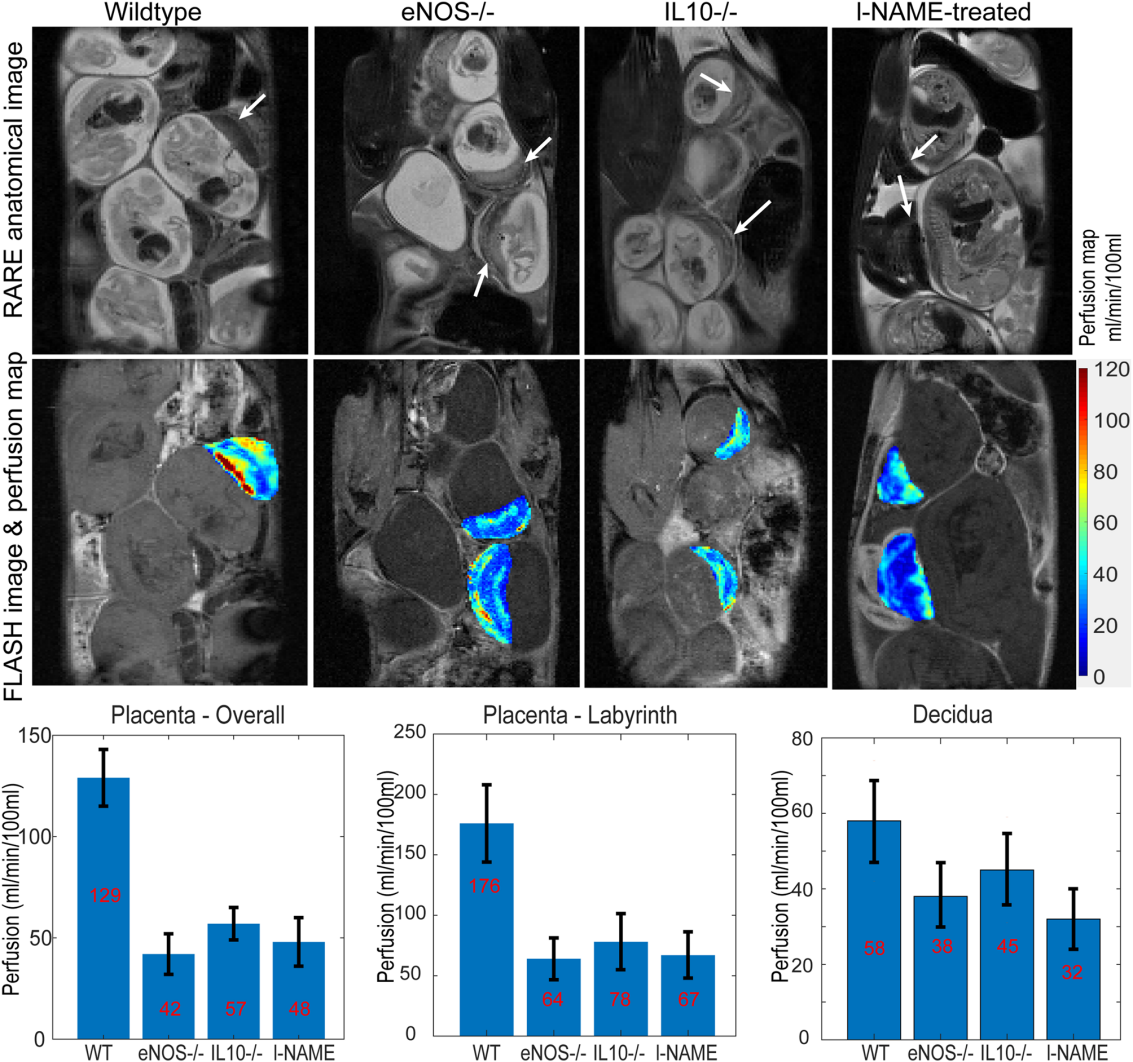
Images and perfusion maps extracted for placentas in wildtype, eNOS^−/−^, IL10^−/−^ and l-NAME-treated mice, calculated using the steepest slope model (see Materials and Methods). The upper row shows T2-weighted anatomical RARE images indicating placentas with white arrows. The middle row shows the FLASH images and perfusion maps extracted for the indicated placentas. The lower row shows mean perfusion coefficients for whole individual placentas, as well as for the segmented labyrinth (fetal side) and decidual compartments. Error bars indicate the range observed in these values over the mice cohorts. Measurements included n = 3 wildtype mice (total number of fetuses = 12), n = 3 eNOS^−/−^ mice (number of fetuses = 15), n = 3 IL10^−/−^ mice (number of fetuses = 13), and two l-NAME-injected mice (number of fetuses = 7). Data were recorded at age E14.5 for the wildtype and knockout mice, and E16.5 for the l-NAME treated one.

The 7 T measurements presented above lacked the sensitivity and resolution needed to reliably analyze the diffusion behavior displayed by the individual placental layers in the various mice models. *In lieu* of this we investigated the picture emerging upon using Gd-DTPA for monitoring the perfusion of the two distinct placental regions arising in contrast-enhanced studies. As described in Supporting Figure S1, these include the labyrinth region proximate to the fetus irrigated by larger material spiral arteries, and the maternal-side decidual region. Figure 7 presents representative images and a summary resulting from monitoring these perfusive phenomena for the various mice models targeted in this study, upon administering the dams with Gd-DTPA tail-vein injections. As has been observed when injecting other Gd-based agents in naïve rodents^33,34^, the labyrinth shows a markedly faster perfusion. This, however, is mainly for the wildtype control animals; for the diseased models, the labyrinth’s perfusion is considerably slower, and not very different from the perfusion observed throughout the rest of the placenta. These differences in DCE persist more or less throughout the pregnancy, and are probably a reflection of a decreased ability of the placentas to effect maternal → fetal transfers in the diseased models.

**Figure 8:**
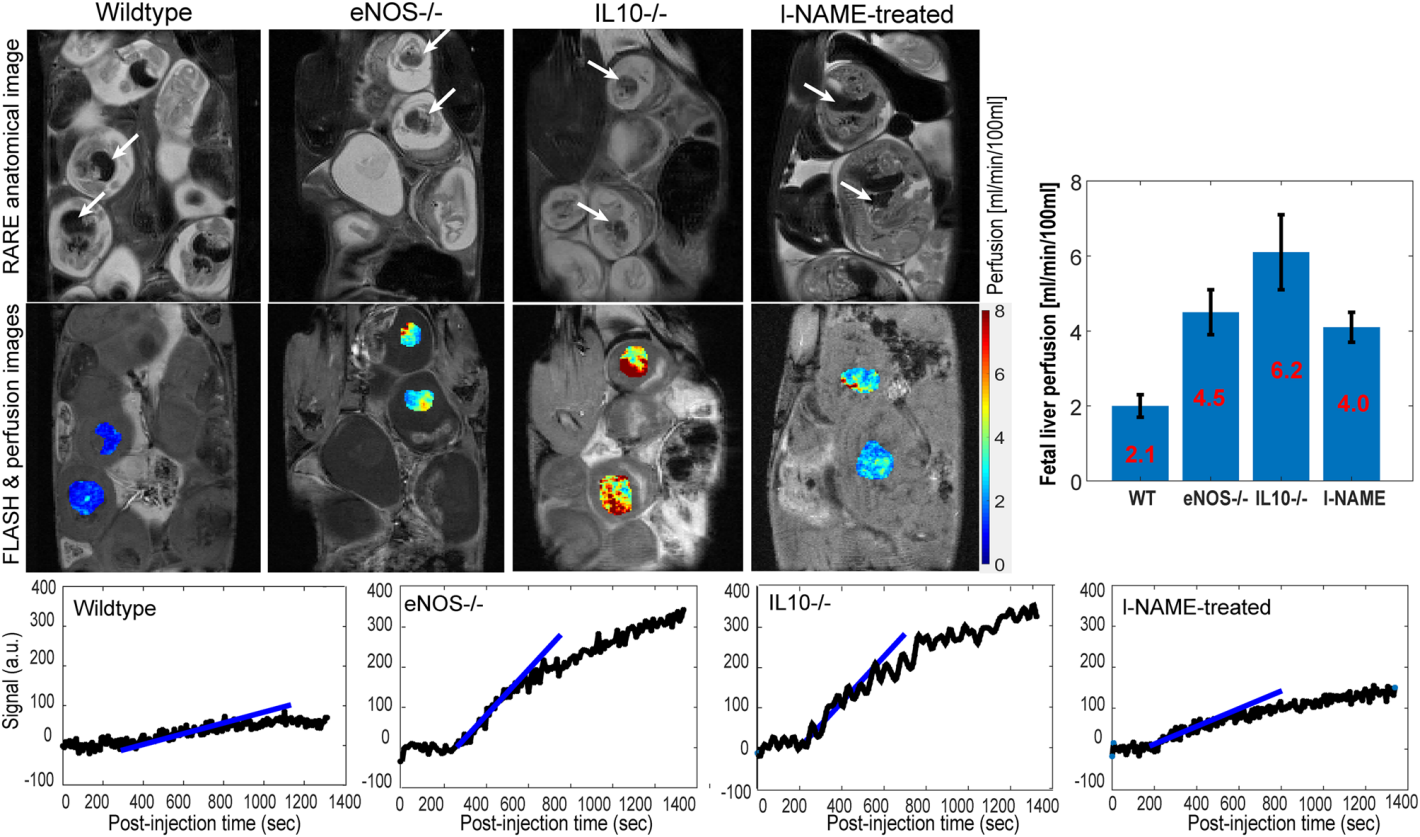
Perfusion maps of selected fetal livers (white arrows) chosen for wildtype, eNOS^−/−^, IL10^−/−^ and post-l-NAME-treated mice, calculated using the initial steepest-slope model (illustrated on the bottom row for selected livers in different animals). The right-most bar graph summarizes these perfusion data for the whole animal cohort—the same cohort as in Fig. 7—suggesting that perfusivity into wildtype mice’s fetal livers is smaller than in the diseased mice models.

The results of these Gd-DTPA-based DCE experiments were evaluated on other portions of the fetoplacental units. Within the fetuses, the sole organ where minor but systematic enhancements could be detected, were the livers (Fig. 8). The average perfusivity for these organs was ca. two orders of magnitude lower than that of the placentas, yet still consistently measurable. Interestingly, the data revealed that by contrast to what was observed in the placentas, the perfusion values in the wildtype mice were significantly smaller than in the diseased animals. We ascribe this to a physiological symptom of the diseased models, as for healthy pregnancies the amount of perfused contrast agent going across the placental barrier and into the fetuses should be minimal.

## Discussion and conclusions

Valuable insight could arise from MRI maps describing how isotropic (and eventually, tensorial) diffusivity, reflect the in vivo morphology of healthy and diseased fetoplacental units, as well as their transport properties. The present study exploited the use of emerging MRI techniques capable of targeting diffusivity in the small and challenging environments posed by murine pregnancy, to evaluate three PE and IUGR models. Differences in the diffusion and perfusion behaviors were observed between wildtype and knockout/treated mice, for fetal brains, for the placentas and their various layers, and for fetal livers which although hypointense in the images and leading to ADC maps of poor sensitivity, became visible under the short echo-time conditions used to collect the DCE data. Most evident among all differences were the alterations in placental diffusivities, which in the knock-out and l-NAME-treated mice models were significantly lower than the average observed for the wildtypes. These differences in transport were echoed by the T1-weighted DCE studies, which also revealed a more facile perfusion for the wildtype mice than the diseased models. DCE’s higher spatial resolution also allowed us to trace distinct differentiations in the labyrinth layers of the various placentas. These complementary descriptions are most likely reflecting the onset of common differences in maternal/fetal transports, where restrictions are associated to IUGR and/or PE. Notable as well was the percolation into the fetal liver that the DCE measurements showed for the gadolinium complex–something that should be discouraged by the fetoplacental barrier. This was hardly evident for the wildtype controls, where ca. two orders of magnitude separated perfusion rates in the fetal livers and in placentas. Gd^2+^ perfusion however was clearly present in the diseased animals, with differences between fetal liver and placental transport were only ~ tenfold. Such anomalous barrier breach could also have long-term influence in post-natal developments.

More subtle were the differences revealed for the brains in the wildtype and diseased animals. Average ADCs for all models were similar, with minor differences erasing away towards the end of the pregnancy. This might be reflecting the onset of the brain sparing process, whereby fetal physiology gives priority to the normal development of the brain and of other vital organs, even if subject to hypoxic, transport or nutritional challenges^37^. Brains in the developmentally-challenged models also showed a noticeable spread in their ADC values, a heterogeneity which could be related residual alterations that may have bearing in post-natal neurostructures^38,39^. These distributions in ADC values could also be reflecting heterogeneities in the expression of the vasoconstriction phenotype among the animals that were analyzed. To explore this possibility the fetoplacental results were revisited on an animal-by-animal basis; Supporting Information Figs. S5 and S6 illustrate their outcome for the brain and placental ADC analyses. As can be seen thereare no meaningful inter-litter variations in what concerns all the placental ADC distributions; neither are there significant variations between the ADC brain distributions observed for the wildtype or l-NAME-treated animals. There are, however, systematic inter-animal biases among the eNOS^−/−^ and the IL10^−/−^ KO mice, which could be partly responsible for the scattering observed for in Fig. 4b among these animals’ brain ADCs. These different degrees of apparent brain maturation could also be of relevance in defining post-natal development.

By probing structure and transport, these ADC and DCE results open new vistas for understanding the physiology of pregnancy in health and disease on preclinical models. Higher-field developmental, functional, metabolic and physiological studies of dam and fetal changes in these and other animal models, are currently in progress^40,41^. Given the non-invasive nature of the novel diffusion methods here described, we also hope they will open new prospects for detecting and better understanding placental disorders at clinical levels. Implementation of these studies are also being planned.

## Supplementary information

Supplementary file 1

## Abbreviations

ADC: Apparent diffusion coefficient
DCE: Dynamic contrast enhanced
EPI: Echo planar imaging
DWI: Diffusion weighted imaging
IUGR: Intrauterine growth restriction
FOV: Field of view
l-NAME: Nitro-l-arginine methyl ester hydrochloride
PE: Preeclampsia
RARE: Rapid acquisition with relaxation enhancement
SPEN: Spatio-temporal encoding
