# Supplementary material for: Diffusion and perfusion MRI of normal, preeclamptic and growth-restricted mice models reveal clear fetoplacental differences
Source: Sci Rep. 2020 Oct 2;10:16380. doi: 10.1038/s41598-020-72885-9 (PMC7532452; doi:10.1038/s41598-020-72885-9)
Supplement: Supplementary file 1 [file 41598_2020_72885_MOESM1_ESM.pdf]

Supporting Information for

Diffusion and perfusion MRI of normal, preeclamptic and growth-restricted mice models reveal clear fetoplacental differences

Qingjia Bao,[1] Ron Hadas,[2] Stefan Markovic,[1] Michal Neeman[2] and Lucio Frydman[1,*]

Departments of [1]Chemical and Biological Physics and [2]Biological Regulation, Weizmann Institute, 7610001 Rehovot, Israel

This section contains additional images and data sets referred to in the manuscript's main text.

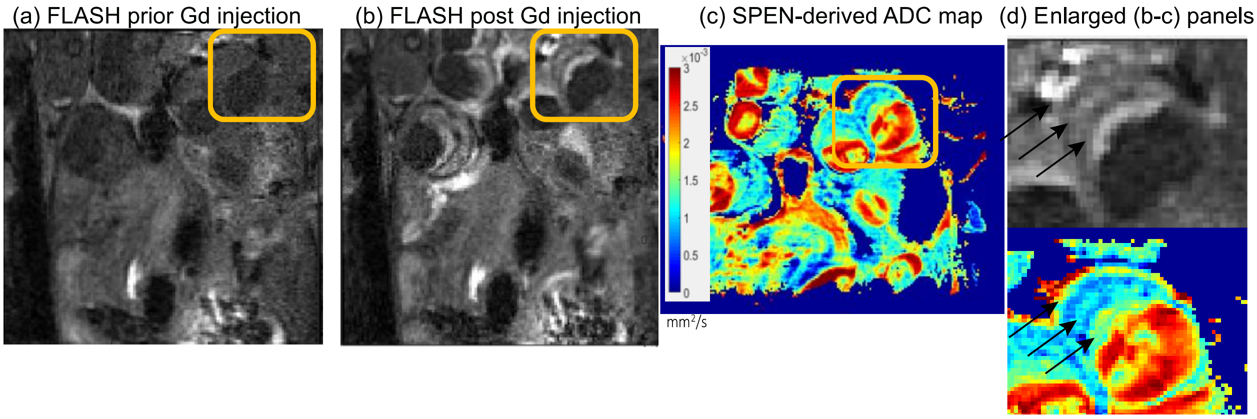

Supporting Figure S1: Assignment of the maternal and fetal layers observed in the ADC maps of mice placentas, based on comparisons between contrast-enhanced and diffusion-derived images for a wildtype animal at day E10.5 of gestation. Prior to BSA-GdDTPA administration the placenta is not visible in the T1-weighted FLASH image (a), but it becomes clearly visible thereafter (b). One of the visible placentas is highlighted by the gold-colored box in this image – acquired 2min after BSA-GdDTPA administration. (c) SPEN ADC maps of the same mouse, highlighting the same placenta. Images in (d) zoomed the gold rectangles containing the placenta in (a)-(c) to 12x12 mm, highlighting with arrows the individual placental layers revealed by the two experiments. FLASH acquisition parameters: TR/TE=60/2.6ms, flip angle=75˚, matrix size = 128×128, FOV=35×35 mm^2 . SPEN ADC mapping parameters: TR/TE = 2000/37ms, FOV=30×30 mm^2, data matrix=160×160, nominal resolution=187μm×187μm×1mm, 5 interleaves, 4 averages, nominal b-values = 0, 750 s/mm^2; three orthogonal diffusion orientations.

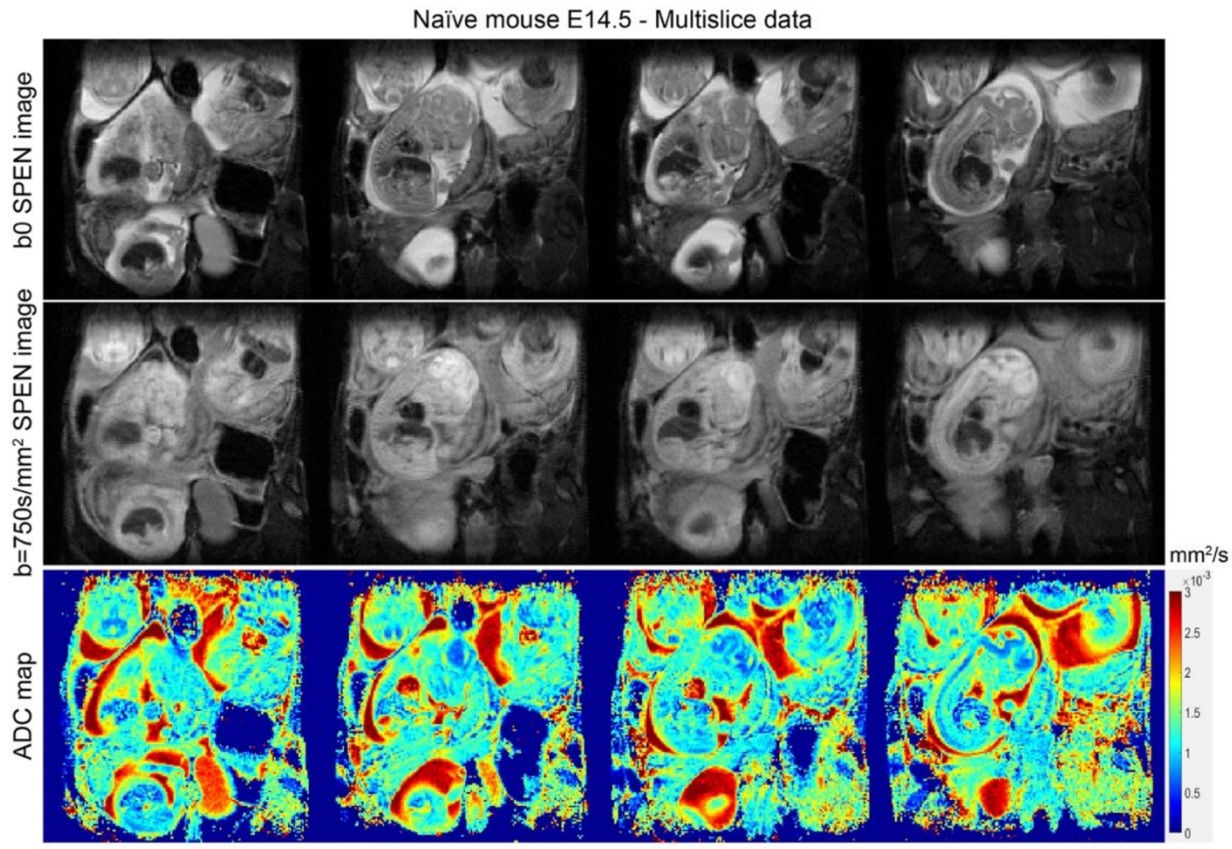

Supporting Figure S2. Multi-slice coronal scans of a pregnant wildtype mouse's abdomen, leading to the ADC maps forming the basis of this study. The sequence used is as described in Materials and Methods, and acquisition parameters as described in Figure 2 of the main text.

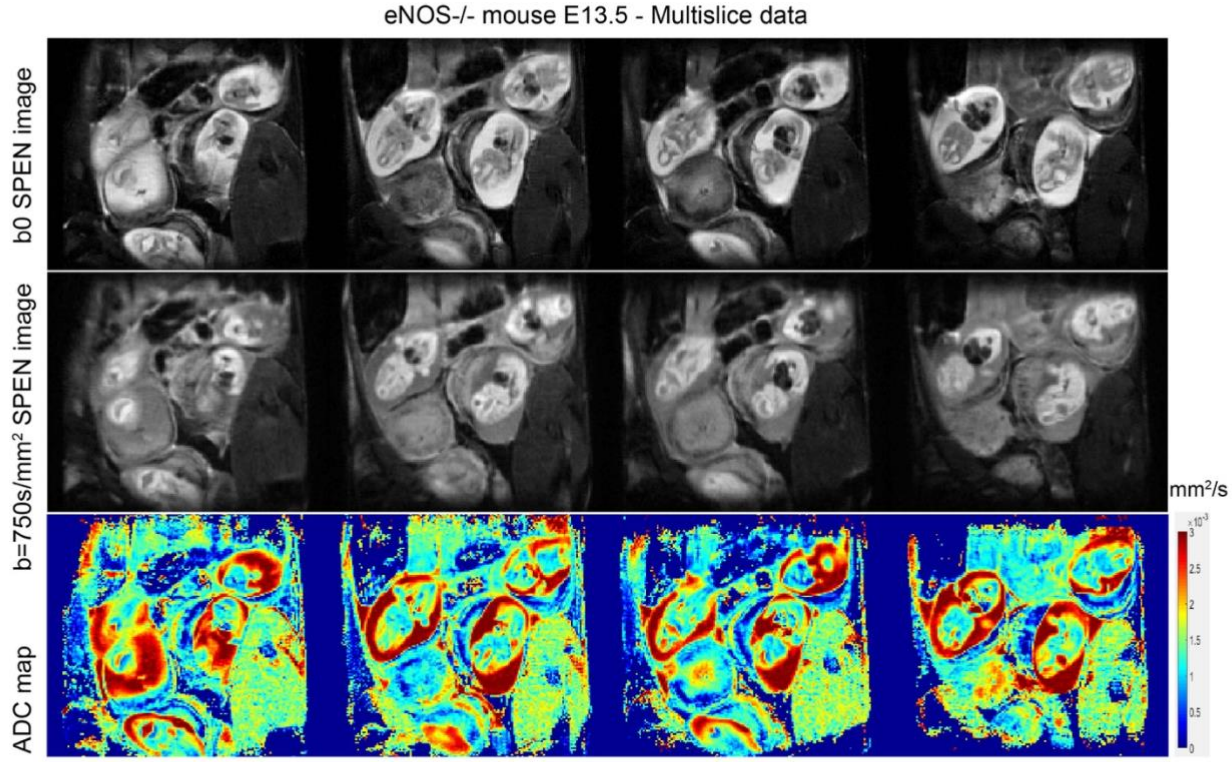

Supporting Figure S3. Idem as in Supporting Figure S2, but for an eNOS-/- deficient animal on day E13.5.

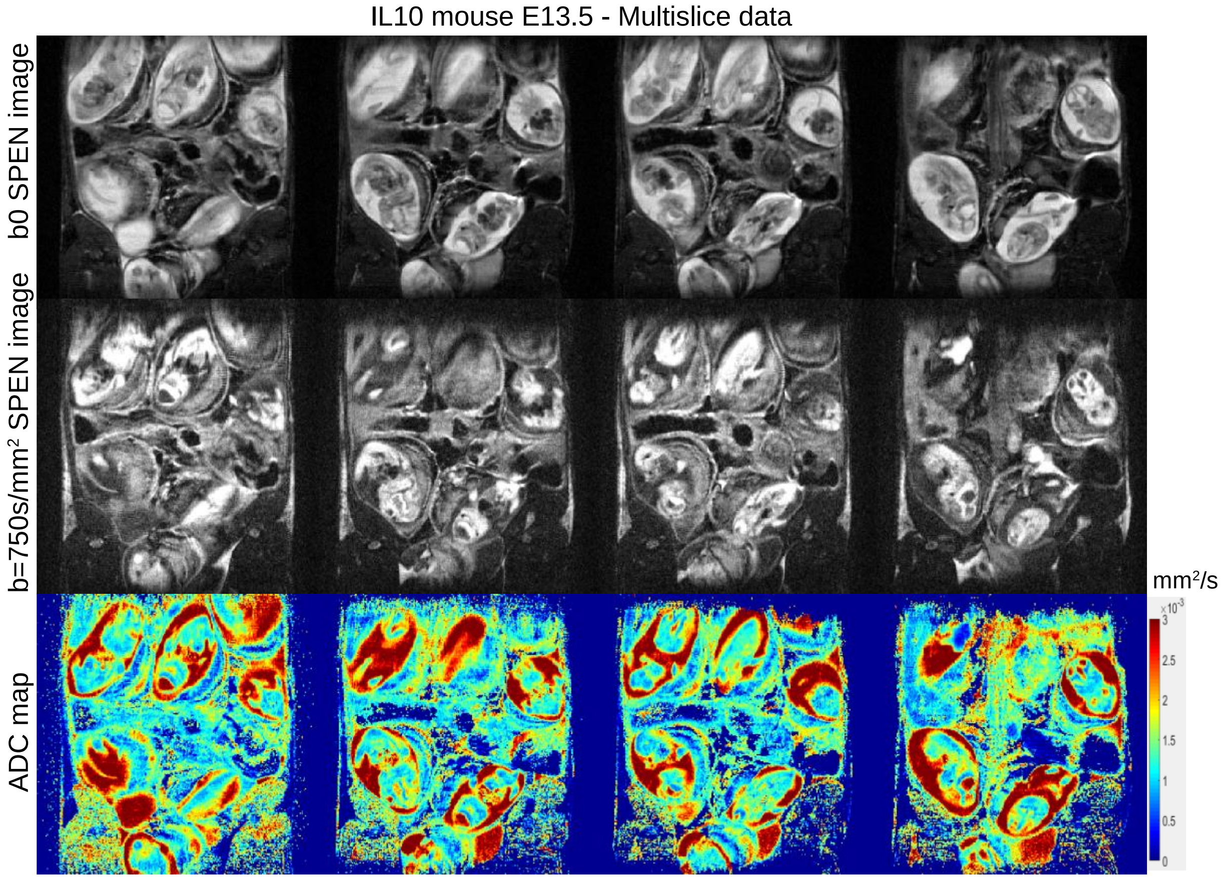

Supporting Figure S4. Idem as in Supporting Figure S3, but for an IL10-/- deficient animal.

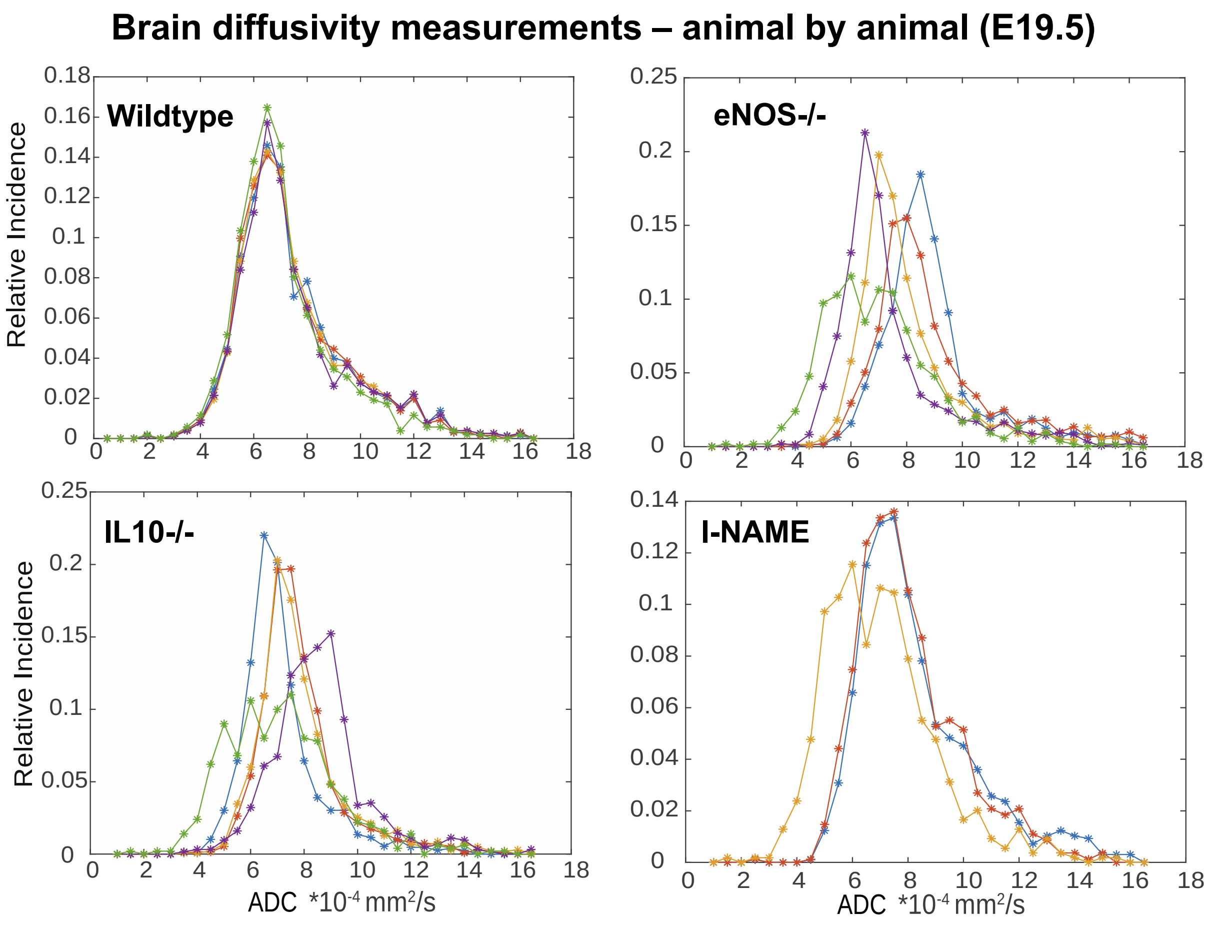

Supporting Figure S5. Fetal brain ADC distributions associated to the full litter of fetoplacental units examined at day E19.5 of the pregnancy, for the different phenotypes specified on top of each graph. Each color represents a different animal within the wildtype, knockout or l_NAME-treated litters; error bars were omitted to simplify the graphs. Notice the center-of-mass displacements observed for the eNOS-/- and IL10-/- cohorts, which could be associated to heterogeneities in the expression of the knockout phenotype and explain at least partly the ADC heterogeneities introduced in Fig. 4b.

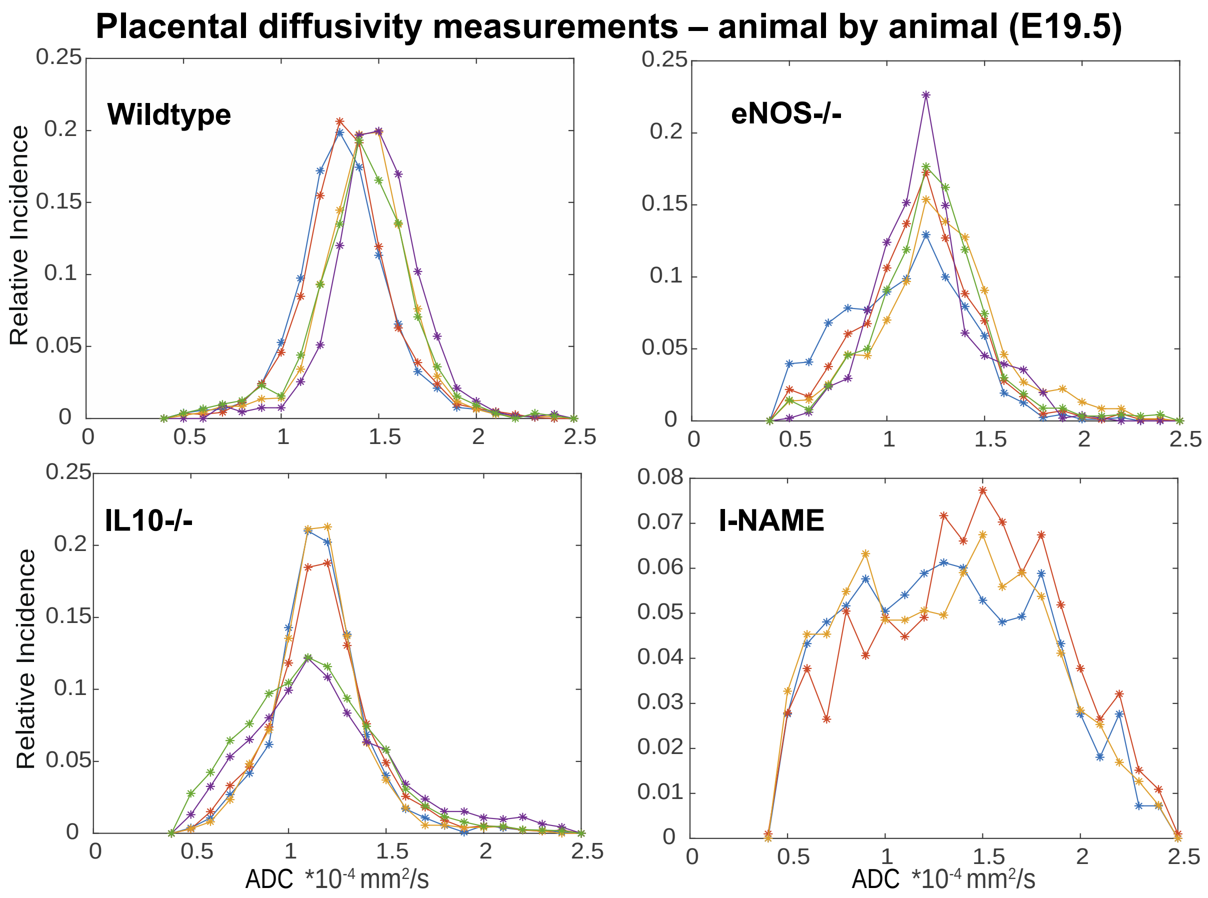

Supporting Figure S6. Idem as presented in Supporting Figure S5, but addressing the ADC distributions of the placentas within the cohorts. Notice that by contrast to what was observed in the brain-related figure, no evident inter-animal biases arise for any of the phenotypes.
